# Vitamin D and Lung Outcomes in Elderly COVID-19 Patients

**DOI:** 10.3390/nu13030717

**Published:** 2021-02-24

**Authors:** Alberto Sulli, Emanuele Gotelli, Andrea Casabella, Sabrina Paolino, Carmen Pizzorni, Elisa Alessandri, Marco Grosso, Diego Ferone, Vanessa Smith, Maurizio Cutolo

**Affiliations:** 1 Laboratory of Experimental Rheumatology and Academic Division of Clinical Rheumatology, Department of Internal Medicine, University of Genova, IRCCS San Martino Polyclinic, 16132 Genova, Italy; emanuele.gotelli@live.it (E.G.); andrea.casabella@unige.it (A.C.); sabrina.paolino@unige.it (S.P.); carmen.pizzorni@unige.it (C.P.); elisa.alessandri@hsanmartino.it (E.A.); mcutolo@unige.it (M.C.); 2 Pneumology Unit, IRCCS San Martino Polyclinic, 16132 Genova, Italy; stilicone73@yahoo.it; 3 Endocrinology Unit, Department of Internal Medicine, University of Genova, IRCCS San Martino Polyclinic, 16132 Genova, Italy; ferone@unige.it; 4 Department of Rheumatology, Ghent University Hospital, Department of Internal Medicine, VIB Inflammation Research Centre Ghent University, 9000 Ghent, Belgium; Vanessa.Smith@UGent.be

**Keywords:** vitamin D, COVID-19, lung involvement, SARS-CoV-2, disease severity

## Abstract

**Background and aim:** Vitamin D deficiency is frequently reported in patients with SARS-CoV-2 infection. The aim of this study was to correlate the 25OH-Vitamin D serum concentrations with clinical parameters of lung involvement, in elderly patients hospitalized for SARS-CoV-2 infection. **Methods:** Sixty-five consecutive COVID-19 patients (mean age 76 ± 13 years) and sixty-five sex- and age-matched control subjects (CNT) were analyzed. The following clinical parameters, including comorbidities, were collected at admission: type of pulmonary involvement, respiratory parameters (PaO_2_, SO_2_, PaCO_2_, PaO_2_/FiO_2_), laboratory parameters (including 25OH-vitamin D, D-dimer, C-reactive protein). **Results:** Significantly lower vitamin D serum levels were found in COVID-19 patients than in CNT (median 7.9 vs. 16.3 ng/mL, *p* = 0.001). Interestingly, a statistically significant positive correlation was observed between vitamin D serum levels and PaO_2_ (*p* = 0.03), SO_2_ (*p* = 0.05), PaO_2_/FiO_2_ (*p* = 0.02), while a statistically significant negative correlation was found between vitamin D serum levels and D-dimer (*p* = 0.04), C-reactive protein (*p* = 0.04) and percentage of O_2_ in a venturi mask (*p* = 0.04). A negative correlation was also observed between vitamin D serum levels and severity of radiologic pulmonary involvement, evaluated by computed tomography: in particular, vitamin D was found significantly lower in COVID-19 patients with either multiple lung consolidations (*p* = 0.0001) or diffuse/severe interstitial lung involvement than in those with mild involvement (*p* = 0.05). Finally, significantly lower vitamin D serum levels were found in the elderly COVID-19 patients who died during hospitalization, compared to those who survived (median 3.0 vs. 8.4 ng/mL, *p* = 0.046). **Conclusions:** This study confirms that 25OH-vitamin D serum deficiency is associated with more severe lung involvement, longer disease duration and risk of death, in elderly COVID-19 patients. The detection of low vitamin D levels also in younger COVID-19 patients with less comorbidities further suggests vitamin D deficiency as crucial risk factor at any age.

## 1. Introduction

Vitamin D plays a central role in bone metabolism, as well as in immune function. Concerning the latter, it regulates the growth and differentiation of several cell types involved in the immune reactivity, displaying anti-inflammatory and immunoregulatory properties [1,2,3]. Therefore, vitamin D may have a key role in mediating the interactions between the innate and adaptive pathways of the immune system [4]. 

Vitamin D serum concentrations are usually lower in elderly people and have been inversely associated with autoimmune inflammatory disease activity and severity, as well as with risk of pulmonary infections [5,6]. Recently, a role for vitamin D deficiency has been reported also in patients with SARS-CoV-2 infection [7,8].

By considering the higher prevalence of COVID-19 in older people, the aim of this study was to correlate, in elderly patients hospitalized for SARS-CoV-2 infection, the 25OH-Vitamin D (25OHD) serum concentrations with clinical parameters of lung involvement.

## 2. Materials and Methods

Sixty-five consecutive COVID-19 patients (mean age 76 ± 13 years, mean disease duration from first symptom 13 ± 13 days, 30 males and 35 females) and sixty-five sex- and age-matched non-COVID control subjects (CNT) were enrolled in the early months of the recent pandemic (March–April 2020) and retrospectively analyzed. All patients needed hospitalization in the COVID-19 dedicated medical unit and were on oxygen therapy for severe respiratory failure due to SARS-CoV-2 infection, but none needed intensive care treatment or intubation at entry. COVID-19 was confirmed in all patients by positive real-time reverse transcriptase polymerase chain reaction on nasal swabs during hospitalization. All patients and controls were Caucasian coming from the same region of the country. Control subjects were outpatients attending the Rheumatology Clinic in the same period. 

The study was conducted in accordance with the principles of the Declaration of Helsinki and Good Clinical Practice and all patient data were collected during the routine medical visits. All the patients gave written informed consent to manage their clinical data and the study was approved by Regional Ethics Committee (ID 10851, 459/2020).

The lung clinical parameters, along with comorbidities and laboratory parameters, were collected at admission. 

The type of pulmonary involvement (localized/mild interstitial lung disease-ground glass pattern, diffuse/severe interstitial lung disease, focal lung consolidation, multiple lung consolidations) was assessed by computed tomography using a high-resolution CT imaging scanner (Siemens Definition Flash, 128 slice, Erlangen, Germany).

Respiratory parameters (PaO_2_, SO_2_, PaCO_2_) were measured by arterial blood gas analysis, and the ratio of arterial oxygen partial pressure to fractional inspired oxygen (PaO_2_/FiO_2_) was calculated to assess the alveolar injury and severity of hypoxemia. Blood samples collected from the radial artery were analyzed by ABL90 FLEX blood gas analyzer (Radiometer, Brønshøj, Denmark).

Laboratory parameters (25OHD, D-dimer, C-reactive protein, ferritin, LDH, blood cell count, calcium, phosphorus, parathyroid hormone, liver and renal function) were assayed by routine standardized assessments in the hospital laboratories, and blood samples were taken at 8 a.m. on the first day of hospitalization. In particular, 25OHD serum levels were measured by standardized chemiluminescence (Liaison 25OH-Vitamin D Total Assay, by DiaSorin, Milan, Italy), and the reported intra and inter assay coefficients of variation (%CV) were 5.4 and 10.6 respectively. Vitamin D serum levels were classified as sufficient, insufficient, deficient, or severely deficient, as suggested by the Endocrine Society guidelines [9].

Duration of hospitalization and global duration of COVID-19 from first symptom until recovery were also recorded. 

Statistical analysis was carried out by StatView software. The Mann–Whitney U test was used to compare unpaired groups of variables, and the Kruskal–Wallis test to compare continuous variables with nominal variables with more than two levels. Possible associations between variables were assessed by Spearman rank correlation, simple, and multiple regression tests. Multiple linear regression models to estimate the associations between 25OHD and outcome measures were performed also after log-transformation of variables, while adjusting for possible confounders. The one-sample Kolmogorov–Smirnov test was used to check the normality assumption of the linear model residuals. Chi-square test was used to compare categorical variables. Any *p* values equal or lower than 0.05 was considered statistically significant; correlation coefficients (r) are also reported throughout the manuscript. The results are reported as median along with interquartile range (IQR).

## 3. Results

Clinical characteristics, comorbidities and baseline clinical parameters of enrolled patients are reported in Table 1 and Table 2. 

Vitamin D serum levels were found significantly lower in COVID-19 patients than in CNT (median 7.9 vs. 16.3 ng/mL, *p*= 0.001) (see Table 3 for further details). No statistically significant difference was observed between male and female subjects (Table 3).

**Figure 1:**
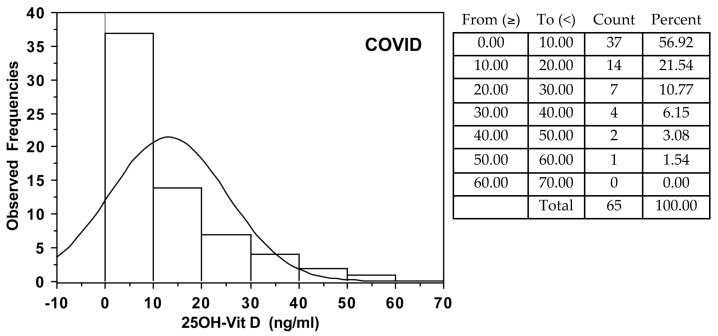

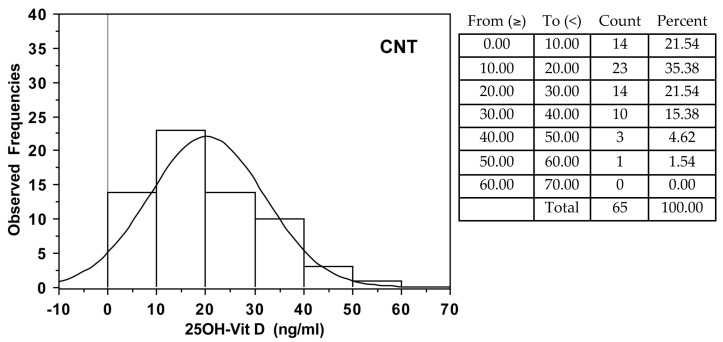
Observed frequencies of 25OH-vitamin D serum levels in COVID-19 patients and control subjects (CNT).

Among COVID-19 patients, vitamin D sufficiency (>30 ng/mL), insufficiency (between 20 and 30 ng/mL), deficiency (between 10 and 20 ng/mL), and severe deficiency (<10 ng/mL) were observed respectively in 11, 11, 21, and 57% of patients. In CNT, the same vitamin D distribution occurred in 21, 21, 36, and 22% of subjects, respectively (Figure 1).

Basal vitamin D serum levels were significantly lower in patients who died during hospitalization, compared to those who survived (median 3.0 vs. 8.4 ng/mL, *p* = 0.046) (Table 3). COVID-19 patients died in relation to the severity of the lung involvement and their comorbidities represented concomitant risk factors.

**Figure 2:**
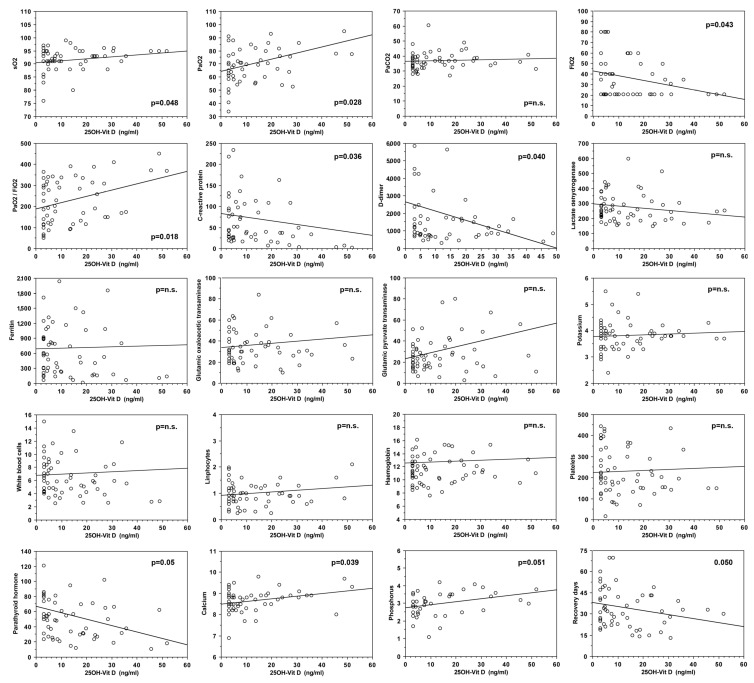
Correlations between 25OH-vitamin D serum levels and clinical parameters in COVID-19 patients.

A statistically significant correlation was observed between vitamin D serum levels and PaO_2_ (*r* = 0.37, *p* = 0.03), SO_2_ (*r* = 0.37, *p* = 0.05), PaO_2_/FiO_2_ (*r* =0.41, *p* = 0.02), while a statistically significant negative correlation was found between vitamin D serum levels and percentage of O_2_ in Venturi Mask (*r* = −0.37, *p* = 0.04) (Figure 2). No correlation was observed between vitamin D serum levels and PaCO_2_.

Furthermore, we fitted three multiple linear regression models to estimate the associations between 25OHD and PaO_2_/FiO_2_, SO_2_, and PaO_2_ while adjusting for age, sex, and number of comorbidities. Since the vitamin D levels were not normally distributed, we modelled the PaO_2_/FiO_2_ ratio with logarithmic transformation and used the log-transformed 25OHD levels as explanatory variable with age, sex and comorbidities as adjustments. The one-sample Kolmogorov–Smirnov test was used to check the normality assumption of the linear model residuals, and the multiple linear regression results are reported in Table 4. 

A statistically significant association between vitamin D serum levels and both PaO_2_/FiO_2_ ratio and PaO_2_ was confirmed. The models show about 17% increase in PaO_2_/FiO_2_ every 1% increase of 25OHD, regardless of age, sex, and comorbidities (Kolmogorov-Smirnov: D = 0.131, *p* = 0.251), as well as for every 1% increase of 25OHD levels, PaO_2_ increases about 5 mmHg. No statistically significant association was detected between SO_2_ and 25OHD after adjustments.

**Figure 3:**
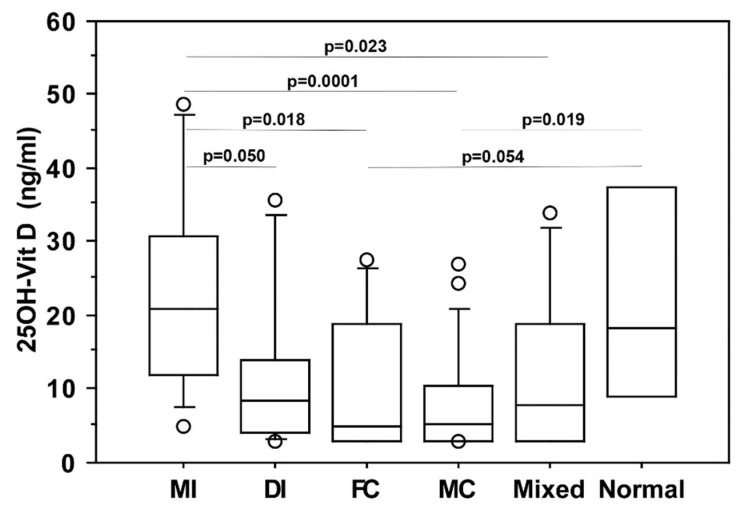
25OH-vitamin D serum levels in COVID-19 patients with different radiological lung involvement. MI = localized/mild interstitial (11 patients), DI = diffuse/severe interstitial (8 patients), FC = focal consolidation (eight patients), MC = multiple consolidations (21 patients), Mixed = mixed abnormalities (13 patients), Normal = absence of radiological involvement (four patients). Data are given as 5th, 10th, 50th (median), 90th, and 95th percentiles. Only statistically significant differences between groups are reported.

A negative association was found between vitamin D serum levels and severity of radiologic pulmonary involvement, as evaluated by CT: in particular, vitamin D was found significantly lower in COVID-19 patients with either multiple lung consolidations (*p* = 0.0001) or diffuse/severe interstitial lung involvement (ground glass pattern) (*p* = 0.05) than in those with mild interstitial lung involvement. Figure 3 reports the statistically significant differences among the groups.

In addition, vitamin D serum levels negatively correlated also with D-dimer (*r* = −0.37, *p* = 0.04), C-reactive protein *(r* = −0.38, *p* = 0.04), and parathyroid hormone (*r* = −0.36, *p* = 0.05) (Figure 2), while serum calcium positively correlated with vitamin D levels *(r* = 0.38, *p* = 0.04). No correlation was observed between vitamin D serum levels and ferritin, LDH, blood cell count, hemoglobin, creatinine, and transaminases. 

Of note, the multiple regression analysis developed to identify possible effects of blood serum parameters (including 25OHD, C-reactive protein, lymphocytes, D-dimer and ferritin as independent variables) on PaO_2_/FiO_2_ ratio (dependent variable) showed vitamin D the only independent variable able to significantly interfere with this clinical parameter (regression coefficient 2.50, *t*-value 2.05, 95% lower 0.05, 95% upper 4.95, *p* = 0.046). 

A negative correlation was also observed between vitamin D serum levels and age of patients in CNT as expected (*r* = −0.49, *p* = 0.0009), but this correlation was not observed in COVID-19 patients *(p* = 0.77).

Finally, lower vitamin D serum levels were found associated with longer global disease duration *(r* = −0.37, *p* = 0.05) (Figure 2).

Multiple linear regressions using age, sex, and number of comorbidities (with vitamin D as the dependent variable) did not identify statistically significant effects of the independent variables on 25OHD level in COVID-19 patients. 

## 4. Discussion

Present investigation underlines that serum vitamin D deficiency might influence the severity of lung involvement and the risk of deaths, in elderly COVID-19 patients hospitalized for SARS-CoV-2 infection.

The results are likely linked to the role played by the biologically active metabolite of vitamin D [1,25(OH)_2_-D] that as steroid hormone is involved in the regulation of growth and differentiation of various immune cell types [3,5,10].

On this basis, the association between vitamin D deficiency and an increased incidence and higher activity of several autoimmune diseases (i.e., systemic lupus erythematosus, rheumatoid arthritis, systemic sclerosis, psoriasis, multiple sclerosis, inflammatory bowel diseases, type 1 diabetes) has been already demonstrated by several reports [2,11,12,13,14,15]. 

On the other hand, low vitamin D levels have been associated with the increased risk of respiratory tract infections and pneumonia, whereas a two-fold increased risk of tuberculosis was observed in patients with vitamin D deficiency, with vitamin D levels predicting the tubercular disease risk in a serum concentration-dependent manner [16,17,18,19]. 

In addition, vitamin D deficiency was found associated to longer acute respiratory infection course, while a recent meta-analysis showed that vitamin D supplementation could prevent respiratory infections [20,21].

Recently, vitamin D deficiency was associated with SARS-CoV-2 infection, in terms of higher risk of disease development, higher disease severity, higher frequency of intensive care unit hospitalization, higher risk of death, thus interfering with the prognosis of COVID-19 [7,8,22,23,24,25,26]. 

SARS-CoV-2 may induce a severe acute respiratory distress syndrome (ARDS) requiring hospitalization, whose main risk factors, besides vitamin D deficiency, are old age, male sex, obesity, hypertension, diabetes, low ambient temperature, and high geographic latitude [26,27]. 

In particular, ageing correlates with progressively lower vitamin D serum levels, as known [28,29]. In our study the mean age of enrolled patients was 76 years: however median vitamin D serum levels were 7.9 ng/mL in COVID-19 patients, significantly lower than that of sex- and age-matched control subjects (16.3 ng/mL). 

Vitamin D deficiency (<20 ng/mL) was also found more frequent in COVID-19 (78% of patients) than in sex- and age-matched controls (57% of subjects). 

Even if the comparison of vitamin D serum levels detected into different studies is quite difficult, due to different cohorts of enrolled patients and various comorbidities that may influence the levels itself, as well as different methodologies, disease severity and age, the results of our study are quite similar to those of a recent study reporting vitamin D serum levels < 50 nmol/L (<20 ng/mL) in 61% of hospitalized patients (mean age 76 years) [26]. In our study 57% of COVID-19 patients showed more severe deficiency with values < 10 ng/mL.

The same authors observed a significantly greater prevalence of vitamin D deficiency (<50 nmol/L) in patients requiring intensive care treatment than in those without (81% of patients) [26]. Similarly, another study reported 25OHD deficiency in 67% of patients with mild SARS-CoV-2 disease, but in 80% of patients requiring mechanical ventilation [22].

A recent systematic review analyzed seven studies on COVID-19 severity, intensive care treatment, and mortality (1368 patients were included) and detected a mean vitamin D level of 22.9 nmol/L (9.16 ng/mL), higher but similar to that of our cohort of patients (7.9 ng/mL) [8). Patients with good prognosis had significantly higher vitamin D levels compared with those with poor prognosis [8]. Additionally, in our cohort of patients, mortality was associated to lower vitamin D serum levels. Globally, these data confirm an important role of vitamin D in COVID-19 outcome.

Among clinical parameters, we included in our study the PaO_2_/FiO_2_ ratio that is commonly used for the diagnosis of alveolar injury (transfusion-related acute lung injury and acute respiratory distress syndrome) and for the assessment of pulmonary disease course and therapy. Practically, the ratio PaO_2_/FiO_2_ measures the severity of hypoxemia in patients with ARDS and has been included into the consensus definition of ARDS itself [30,31].

A low PaO_2_/FiO_2_ ratio was detected as an independent risk factor for death in COVID-19 patients [32]. Our study detected a statistically significant positive correlation between 25OHD serum levels and PaO_2_/FiO_2_ values (low vitamin D values were associated to low PaO_2_/FiO_2_ ratio). This observation is in line with the results of another study reporting a high prevalence of hypovitaminosis D in COVID-19 patients with low PaO_2_/FiO_2_ ratio [33].

How vitamin D interferes with COVID-19 progression is not completely understood. The innate immune system represents the first line of defense against viruses depending on constitutive expression of pattern recognition receptors, like toll-like receptors. 1,25(OH)_2_-D plays an anti-viral role, regulating the inflammatory response by modulating toll-like receptor expression and NK cell function, and suppressing over-expression of pro-inflammatory cytokines [34]. 1,25(OH)_2_-D enhances the defense also by inducing antimicrobial peptide release, like cathelicidin that lead to viral destruction and clearance and facilitates the recruitment of monocytes, macrophages, neutrophils and dendritic cells. Therefore, 1,25(OH)_2_-D may regulate the innate/adaptive responses and may interfere with the maturation of dendritic cells and their ability to present antigen to T-cells, shifting the T cell profile from the pro-inflammatory Th1 and Th17 subsets to Th2 and Treg subsets, thus inhibiting the pro-inflammatory processes [10]. 

Besides the immunomodulatory and anti-viral effects, 1,25(OH)_2_-D modulates the renin–angiotensin system that also plays a pivotal role in the pathogenesis of COVID-19. ACE2 seems the main host cell receptor that mediates the infection by SARS-CoV-2: the virus attaches to ACE2 through its spike glycoprotein to enter the cell, thus reducing the expression of ACE2 [35,36,37,38,39]. Vitamin D suppresses renin at the transcriptional level and consequently angiotensin expression, and increases ACE2 expression, possibly restoring the physiological concentration of ACE2 downregulated by the virus [40,41,42,43].

In the lung, several alveolar cell types express the ACE2 receptor. These cells play an important role in producing surfactant, able to regulate the alveolar surface tension. SARS-CoV-2 can infect the alveolar cells by ACE2 binding and suppress the production of surfactant. The loss of alveolar cells results in lung damage and respiratory insufficiency due to the loss of pulmonary surfactant [44]. This damage might be prevented by vitamin D, as in vitro and in vivo studies have shown that 1,25(OH)_2_-D induces type II pneumocyte proliferation and surfactant synthesis in the lungs [45,46,47]. Our clinical data are therefore supported by molecular mechanisms. Vitamin D exerts also vasoprotective effects, while vitamin D deficiency represents a risk factor for endothelial dysfunction [48]. Interestingly, vitamin D is involved in the regulation of the thrombotic pathways, and vitamin D deficiency is associated with a higher risk of thrombotic events [49]. As known, patients with COVID-19 frequently suffer from microthrombotic complications, which may contribute to worse lung disease and death. The main autopsy histological findings report a sequential alveolar damage, mainly characterized by focal capillary microthrombosis [50,51]. This clinical complication may be laboratory investigated by D-dimer dosage [52]. High D-dimer levels are often detected in COVID-19 patients, and are significantly associated with the risk of mortality [53]. Our study confirms a statistically significant negative correlation between 25OHD serum levels and D-dimer values, as recently reported also by another study [54]. 

Finally, it must be said that hypovitaminosis D has been hypothesized to be the consequence, rather than the cause, of chronic inflammatory diseases, as well as current data are not yet sufficient to demonstrate a definite role for vitamin D in the modulation of adaptive immune system in humans [55,56]. However, it is biologically plausible that calcitriol may exert immunomodulatory effects, also in COVID-19 patients, by regulating both innate and adaptive immunity [10,57], as the lung epithelium was demonstrated to be an important target tissue for calcitriol [45]. Furthermore, in a clinical case-series report, high dose vitamin D supplementation improved clinical recovery in COVID-19 patients, evidenced by decrease in inflammatory biomarker status, lower oxygen requirements and less days of hospitalization [58]. However, a recent study including UK Biobank subjects did not evidence a potential link between vitamin D concentrations and risk of COVID-19 infection [25]. 

This study has some limitations.

The number of patients analyzed in this retrospective cross-sectional study is small, and included only elderly patients needing hospitalization and oxygen therapy due to severe respiratory failure in SARS-CoV-2 infection, but it did not include either patients with mild disease nor patients needing intensive care treatment or ventilation. Furthermore, due to small number of enrolled patients and large data variability, the sample size was not adequately powered to rule out the variation expected in the main outcome parameters: for this reason the correlation coefficients are relatively small. Therefore, robustly designed randomized clinical trials including a larger number of patients are needed, but are likely to further confirm these preliminary data. 

Enrolled patients did not perform ventilation/perfusion lung scan along with contrast enhanced computed tomography to investigate pulmonary microembolism/thrombosis, and this does not permit to surely associate the D-dimer abnormalities with the clinical condition. However, pulmonary embolism has been described in up to 37% of COVID-19 patients by radiological examinations and in 79% of patients after autopsy [59,60]. 

COVID-19 patients showed several different comorbidities that might interfere with some results of the study, however also sex- and age-matched healthy subjects had similar comorbidities, as reported in Table 1. Of note, the detection of the disease also in younger patients with less comorbidities, further suggests vitamin D deficiency as crucial risk factor at any age. Therefore, vitamin D supplementation is recommended for the prevention and treatment of any age SARS-CoV-2 infection and should be matter of large controlled studies [61,62]. In our cohort, 34% of patients and 68% of controls were supplemented with vitamin D. 

Several other studies have also linked low levels of serum vitamin D to SARS-CoV-2 infection [63]. However, our study clearly reports the associations between lung, clinical and laboratory parameters (including 25OHD levels) in elderly patients hospitalized for COVID-19, focusing on lung involvement. Accordingly, the mean age of analyzed patients was very high and this did not permit further comparison of vitamin D levels between COVID-19 patients and controls by stratifying different ages. 

It is already known that vitamin D serum level is usually low in patients with chronic inflammation or infections (including SARS-CoV-2), but this was previously demonstrated in younger people. This study reports and confirms similar findings also in older COVID-19 patients.

## 5. Conclusions

Vitamin D deficiency is a risk factor for infections as well as for insufficient innate immune system reactivity, therefore the winter time testing for serum vitamin D status (lowest levels expected) should be mandatory as part of regular health check status. The test is even more mandatory in patients with chronic diseases, where we expect vitamin D deficiency during all the year and an increased burden of risk factors for further disease severity and even mortality. Deficiency must be treated with vitamin D administration until reaching the optimal range of 40–60 ng/mL during all the year.

In conclusion, this study confirms that vitamin D deficiency is associated with more severe lung involvement, longer disease duration, and risk of death in elderly COVID-19 patients.

## Figures and Tables

**Table 1:** Clinical characteristics and comorbidities of enrolled subjects. *p* = statistical significance.

	COVID-19	CONTROLS	*p*
Age (years, mean ± SD)	76 ± 13	76 ± 13	0.814
Disease duration (days, mean ± SD)	13.1 ± 13	-	-
Male/Female	30/35	30/35	-
Body mass index (kg/m^2^)	26.3	25.4	0.634
Ethnicity: Caucasian	65/65	65/65	0.931
Died during hospitalization	10/65	-	-
Recovery time (days, mean ± SD)	33 ± 14	-	-
Smoking status	4/65	4/65	0.891
Arterial hypertension	35/65 (54%)	36/65 (55%)	0.281
Previous cardiac/cerebral ischemic vasculopathy	14/65 (21%)	6/65 (11%)	0.357
Neoplasms	11/65 (16%)	5/65 (8%)	0.091
Recent hip or vertebral fracture	12/65 (18%)	11/65 (17%)	0.916
Diabetes	10/65 (15%)	9/65 (14%)	0.737
Chronic atrial fibrillation	11/65 (17%)	11/65 (17%)	0.492
Chronic obstructive pulmonary disease	8/65(12%)	3/65 (3%)	0.213
Chronic kidney disease	5/65 (8%)	4/65 (6%)	0.652
Dysthyroidism	4/65 (6%)	5/65 (8%)	0.458
Colic diverticulosis	5/65 (8%)	2/65 (3%)	0.223
Chronic arthritis (rheumatoid or psoriatic)	2/65 (3%)	7/65 (11%)	0.049
Epilepsy	2/65 (3%)	4/65 (6%)	0.296
Allergic asthma	1/65 (2%)	2/65 (3%)	0.414
Liver cirrhosis	1/65 (2%)	3/65 (5%)	0.243
Hepatitis B infection	1/65 (2%)	1/65 (2%)	0.892
No comorbidities	7/65 (11%)	10/65 (15%)	0.490
One comorbidity	17/65 (26%)	19/65 (29%)	0.749
Two comorbidities	24/65 (37%)	22/65 (34%)	0.647
Three comorbidities	13/65 (20%)	11/65 (17)	0.547
Four or more comorbidities	4/65 (6%)	3/65 (5%)	0.616
Vitamin D supplementation	22/65 (34%)	44/65 (68%)	0.015

**Table 2:** Baseline clinical parameters of COVID-19 patients. Values are reported as median and interquartile range (IQR).

	COVID-19	Normal Range
SO_2_ (%)	92 (4)	95–99
PaO_2_ (mmHg)	69 (20)	83–108
PaCO_2_ (mmHg)	37 (7)	35–48
FiO_2_ (%)	31 (36)	21
PaO_2_/FiO_2_	211 (199)	>300
D-dimer (mcg/L)	1078 (1071)	0–500
C-reactive protein (mg/L)	39 (78)	0–5
Ferritin (mcg/L)	562 (746)	30–400
LDH—lactate dehydrogenase (UI/L)	259 (117)	135–225
White blood cells (×10^9^/L)	5.9 (4.2)	4.5–9.8
Lymphocytes (×10^9^/L)	0.9 (0.6)	1.1–4.8
Haemoglobin (g/L)	11.3 (2.9)	12–17.5
Platelets (×10^9^/L)	218 (166)	130–430
GOT—glutamic oxaloacetic transaminase (UI/L)	34 (21)	0–40
GPT—glutamic-pyruvate transaminase (UI/L)	29 (21)	0–40
Creatinine (mg/dL)	0.9 (0.4)	0.6–1.0
Calcium (mg/dL)	8.7 (0.5)	8.5–11.0
Phosphorus (mg/dL)	3.2 (1.1)	2.5–4.5
PTH—parathyroid hormone (ng/L)	51 (40)	6.5–36.8

**Table 3:** Vitamin D serum levels in COVID-19 patients and control subjects (CNT). 25OH-vitamin D values (ng/mL) are reported as median and interquartile range (IQR).

	COVID-19	CNT	Statistical Significance
All subjects	7.9 (15)	16.3 (19)	*p* = 0.001
Died/Survived	3.0 (8)/8.4 (18)	-	*p* = 0.046
Male	7.0 (12) *	13.6 (20) *	*p* = 0.021
Female	9.3 (20) *	18.3 (19) *	*p* = 0.0049

* male vs. female *p* = n.s.

**Table 4:** Multiple linear regression results showing the statistically significant associations between vitamin D serum levels (25OHD) and both PaO_2_/FiO_2_ ratio and PaO_2_.

Outcome	Predictors	Beta (95% CI)	*p* Value
**PaO_2_/FiO_2_ (log)**	25OHD (ng/mL) (log)	0.17 (0.01, 0.33)	0.033
	Sex (female)	−0.02 (−0.31, 0.27)	0.889
	Age (years)	−0.00 (−0.01, 0.01)	0.812
	Comorbidities	−0.07 (−0.21, 0.06)	0.272
**SO_2_ (%)**	25OHD (ng/mL) (log)	0.97 (−0.66, 2.60)	0.240
	Sex (female)	−2.38 (−5.37, 0.61)	0.116
	Age (years)	0.02 (−0.09, 0.13)	0.713
	Comorbidities	−1.08 (−2.48, 0.31)	0.126
**PaO_2_ (mmHg)**	25OHD (ng/mL) (log)	5.18 (0.83, 9.52)	0.021
	Sex (female)	−10.3 (−18.2, −2.3)	0.012
	Age (years)	−0.03 (−0.33, 0.27)	0.823
	Comorbidities	−1.37 (−5.06, 2.32)	0.459

## Data Availability

The datasets generated and/or analyzed during the current study are not publicly available for ethical and privacy reasons, but are available from the corresponding author on reasonable request.
